# Inflammatory Bowel Disease: How Effective Is TNF-α Suppression?

**DOI:** 10.1371/journal.pone.0165782

**Published:** 2016-11-08

**Authors:** Wing-Cheong Lo, Violeta Arsenescu, Razvan I. Arsenescu, Avner Friedman

**Affiliations:** 1 Department of Mathematics, City University of Hong Kong, Hong Kong, Hong Kong SAR; 2 Armedica LLC, Dublin, Ohio, United States of America; 3 Digestive Health Institute, Inflammatory Bowel Diseases Center of Excellence Morristown Medical Center, Morristown, New Jersey, United States of America; 4 Mathematical Biosciences Institute, Department of Mathematics, The Ohio State University, Columbus, Ohio, United States of America; National Institutes of Health, UNITED STATES

## Abstract

Crohn’s Disease (CD) results from inappropriate response toward commensal flora. Earlier studies described CD as a Th1 mediated disease. Current models view both phenotypes as a continuum of various permutations between Th1, Th2 and Th17 pathways compounded by a range of Treg disfunctions. In the present paper, we develop a mathematical model, by a system of differential equations, which describe the dynamic relations among these T cells and their cytokines. The model identities four groups of CD patients according to up/down regulation of Th1 and Th2. The model simulations show that immunosuppression by TNF-α blockage benefits the group with Th1^High^/Th2^Low^ while, by contrast, the group with Th1^Low^/Th2^High^ will benefit from immune activation.

## Introduction

Inflammatory Bowel Diseases (IBD), Crohn’s Disease (CD) and Ulcerative Colitis (UC) result from an inappropriate immune response toward commensal flora [1]. Genome wide association studies (GWAS) indicated that the majority of IBD susceptibility loci belong to immunoregulatory networks [2, 3].

Patients with inflammatory bowel diseases have elevated levels of circulating and gut mucosal cytokines [1]. Downstream signaling from these inflammatory mediators, through Janus kinase (JAK) and signal transducers and activators of transcription (STAT) proteins, activate transcription factors T-bet, GATA3, Foxp3 and RORγt [3]. A complex network of regulatory feedback loops involving these cytokines and their targets, are responsible for the polarization of naïve T cells into specific T helper cells: Th1, Th2, Th17 and Treg [3, 4].

Earlier animal and human studies described CD as a Th1 mediated [5]. Current models view both phenotypes as a continuum of various permutations between the Th1, Th2, and Th17 pathways compounded by a range of Treg dysfunctions [4].

The development of current biological therapies in CD, mirrors our understanding of immune regulatory pathways. Unfortunately the clinical triage of CD patients, based on Montreal classification, cannot identify relevant immune targets in a given patient [6]. Thus our treatments are inherently a trial and error approach. Pretreatment knowledge of the relevant gut mucosal immune dysfunction in a given patient would significantly improve the risk/benefit balance and open the way toward personalized medical care. We have previously developed a theoretical model that described the relationship between Th1, Th2 and Treg circuits in patients with CD [7]. Our current study expands this model to include the Th17 pathway which plays an important role in inhibiting Treg cell differentiation that associated with autoimmune disorders and inflammation [8, 9]. The levels of Th17 cell transcription factor and related cytokines have been collected in our clinical data to provide better predictions of disease outcome through our new model. Furthermore, based on patients’ data, it allowed us to simulate the effect of TNF-α suppression in a cohort of patients with CD, and thus identify distinct groups which will benefit from TNF-α suppression and groups which may benefit from immune activation rather than immune suppression.

## Materials and Methods

### Mathematical model

**Fig 1:**
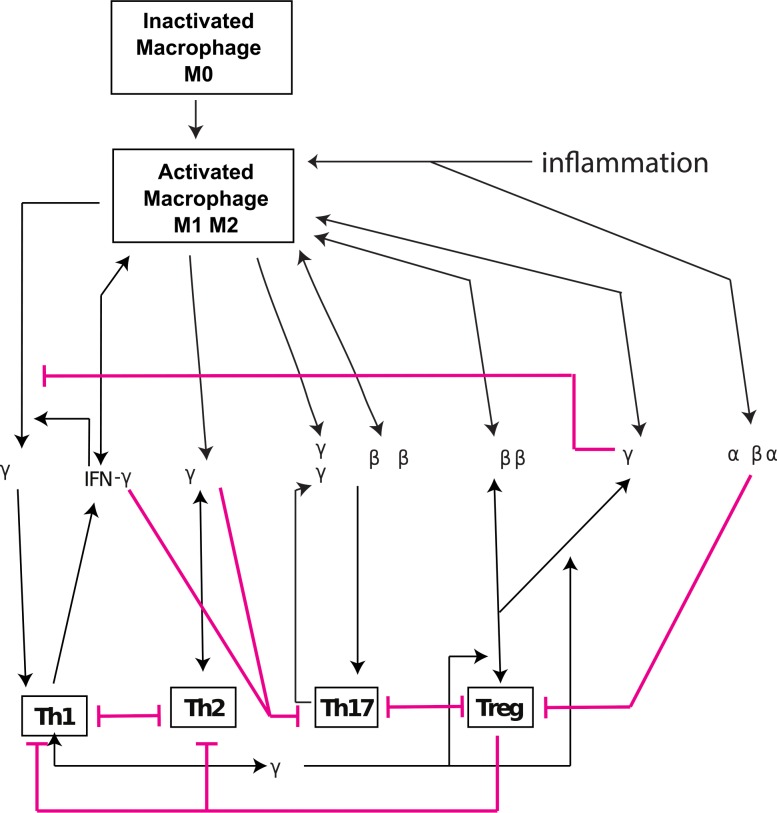
Schematic diagram of immune system with application to inflammatory bowel disease (IBD).

A system of differential equations was developed based on the network that incorporates cytokines and transcriptions factors relevant to Th1, Th2, Th17 and regulatory T cells pathway as shown in Fig 1. The relative concentrations of either immune cells or inflammatory mediators were defined in g/cm^3^ and were based on a theoretical density in a cm^3^ of tissue. The variables (concentrations) included in the model are listed in Table 1.

**Table 1:** List of the variables included in the model.

*M*_*1*_: activated M1 macrophages	*T*_*1*_: Th1 cells
*M*_*2*_: activated M2 macrophages	*T*_*2*_: Th2 cells
*M*: total macrophages (*M*_*1*_ *+ M*_*2*_)	*T*_*17*_: Th17 cells
*I*_*α*_: TNF-α	*I*_*β*_: TGF-β
*I*_*γ*_: IFN-γ	*I*_*2*_: IL-2
*I*_*4*_: IL-4	*I*_*6*_: IL-6
*I*_*10*_: IL-10	*I*_*12*_: IL-12
*I*_*21*_: IL-21	

#### Differential equations for cytokines

IFN-γ is produced by Th1 cells and activates M1 macrophages [10]. In order to derive an equation which expresses the dynamics of *I*_*γ*_, we denote by *dI*_*γ*_ the change in *I*_*γ*_ over a small time interval *dt*, and compute the quotient *dI*_*γ*_*/dt* (the derivative of *I*_*γ*_ with respect to *t*, if *dt* is infinitesimally small). If *I*_*γ*_ is produced by Th1 cells and the production rate coefficient for IFN-γ by Th1 cells is *ν*_*γ1*_, then, at a given Th1 cell density *T*_*1*_, we can write *dI*_*γ*_*/dt = ν*_*γ1*_*T*_*1*_. Similarly, the production of this cytokine by activated macrophages would be described by the differential equation *dI*_*γ*_*/dt = ν*_*γM*_*M*_*1*_. We finally have to account for the fact that IFN-γ tissue concentration decays at some known rate *δ*_*γ*_. Therefore the final formula for the dynamics of *I*_*γ*_ is given by the differential equation:

$$\frac{dI_{\gamma }}{dt}=\underset{\text{Production}}{\underset{︸}{v_{\gamma M}M_{1}+v_{\gamma 1}T_{1}}}-\underset{\text{Decay}}{\underset{︸}{\delta_{\gamma }I_{\gamma }}}.$$ (1)

Similar differential equations were established for IL-2, IL-4, IL-6, IL-21, TGF-β and TNF-α.

IL-2 is secreted by Th1 cells [11, 12], so we have

$$\frac{dI_{2}}{dt}=\underset{\text{Production}}{\underset{︸}{v_{21}T_{1}}}-\underset{\text{Decay}}{\underset{︸}{\delta_{2}I_{2}}}.$$ (2)

Th2 cells and M2 macrophages produce IL-4 during inflammation [4, 10, 13]; hence

$$\frac{dI_{4}}{dt}=\underset{\text{Production}}{\underset{︸}{v_{4M}M_{2}+v_{42}T_{2}}}-\underset{\text{Decay}}{\underset{︸}{\delta_{4}I_{4}}}.$$ (3)

IL-21 is produced by Th17 cells [14, 15], so that

$$\frac{dI_{21}}{dt}=\underset{\text{Production}}{\underset{︸}{v_{2117}T_{17}}}-\underset{\text{Decay}}{\underset{︸}{\delta_{21}I_{21}}}.$$ (4)

IL-6 and TNF-α are produced by M1 [14, 15] and Th1 cells also produce TNF-α [16]; hence, the equations of *I*_*6*_ and *I*_*α*_ are

$$\frac{dI_{6}}{dt}=\underset{\text{Production}}{\underset{︸}{v_{6M}M_{1}}}-\underset{\text{Decay}}{\underset{︸}{\delta_{6}I_{6}}},$$ (5)

$$\frac{dI_{\alpha }}{dt}=\underset{\text{Production}}{\underset{︸}{v_{\alpha M}M_{1}+v_{\alpha 1}T_{1}}}-\underset{\text{Decay}}{\underset{︸}{\delta_{\alpha }I_{\alpha }}}.$$ (6)

TGF-β is mainly expressed from M2 macrophages and Treg cells [4, 10, 17]; hence we have

$$\frac{dI_{\beta }}{dt}=\underset{\text{Production}}{\underset{︸}{v_{\beta M}M_{2}+v_{\beta r}T_{r}}}-\underset{\text{Decay}}{\underset{︸}{\delta_{\beta }I_{\beta }}}.$$ (7)

Cytokine IL-10 can be the product of both M2 macrophages and Treg cells [4, 10, 17], and IL-2 further increases IL-10 production by Treg cells [18]. IL-2 binds its cognate receptors and it is rapidly internalized. Allowing for the receptor recycling rate (*n*_*2r*_) and maximum amount of IL-2 bound at any given time, we can write an equation that links this rate to the tissue concentration of IL-2 (*I*_*2*_) and Treg (*T*_*r*_): *n*_*2r*_ (*I*_*2*_*/*(*ζ*_*2*_ *+ I*_*2*_)) *T*_*r*_ where *ζ*_*2*_ is a constant. Therefore the differential equation that incorporates IL-10 secretion from macrophages, Treg cells and IL-2 contribution can be written as:

$$\frac{dI_{10}}{dt}=\underset{\text{Production}}{\underset{︸}{v_{10M}M_{2}+v_{10r}\left[1+n_{2r}\left(\frac{I_{\gamma }}{\zeta_{2}+I_{2}}\right)\right]T_{r}}}-\underset{\text{Decay}}{\underset{︸}{\delta_{10}I_{10}}}.$$ (8)

IL-12 is produced by M1 macrophages, and IL-10 can suppress its expression [19]. The IL-10 mediated suppression can be described using the expression (1+ *I*_*10*_*/ζ*_*10*_*)* where *ζ*_*10*_ is a constant specific to IL-10 activation rate. Thus the differential equation for the net IL-12 production becomes:

$$\frac{dI_{12}}{dt}=\underset{\text{Production}}{\underset{︸}{v_{12M}\frac{M_{1}}{1+I_{10}/\zeta_{10}}}}-\underset{\text{Decay}}{\underset{︸}{\delta_{12}I_{12}}}.$$ (9)

#### Differential equations for macrophages

The specific polarization of naïve T cells toward Th1, Th2, Th17 and Treg phenotypes is supported by cytokines produced by activated macrophages [4]. Activated macrophages fall into two major groups, M1 and M2. Increased tissue concentration of TNF-α in IBD patients can lead undifferentiated M0 macrophages to polarize to M1 macrophages, classically activated macrophages [20]. M1 macrophages can also be derived from M2 in the presence of high levels of IFN-γ, a proinflammatory cytokine [21]. In contrast, M2 (alternatively activated macrophages) are induced by anti-inflammatory cytokines such as IL-10 and TGF-β [21]. The following factors were considered when obtaining a differential equation for M1 macrophage phenotype formation: concentration of tissue factors (*f*_1_) that contribute to macrophage polarization; tissue concentrations of TNF-α (*I*_*α*_*)*, IFN-γ (*I*_*γ*_) and TGF-β (*I*_*β*_); cytokine specific rates of macrophage activation (*σ*_*Mα*,_ *σ*_*Mβ*,_ *σ*_*Mγ*_) and transition between M1 and M2 phenotypes (*σ*_*Mβ*_, *σ*_*Mγ*_), and macrophage apoptosis (decay) rate (*μ*_*M*_). The differential equation for M1 is given by

$$\frac{dM_{1}}{dt}=\underset{\text{M}0\to M1\;\text{by TNF-}\alpha }{\underset{︸}{\left(f_{1}+\sigma_{M\alpha }\frac{I_{\alpha }}{\zeta_{\alpha }+I_{\alpha }}\right)M_{0}}}-\underset{\text{M}1\to M2\;\text{by TGF-}\beta }{\underset{︸}{\sigma_{M\beta }\frac{I_{\beta }}{\zeta_{\beta }+I_{\beta }}M_{1}}}+\underset{\text{M}2\to M1\;\text{by IFN}\gamma }{\underset{︸}{\sigma_{M\gamma }\frac{I_{\gamma }}{\zeta_{\gamma }+I_{\gamma }}M_{2}}}-\underset{\text{Decay}}{\underset{︸}{\mu_{M}M_{1}}}.$$ (10)

Similarly, undifferentiated M0 macrophages polarize to M2 macrophages under the influence of IL-10 [22], while M1 convert to M2 macrophages by TGF-β [21], so that the complete formula for *dM*_*2*_*/dt*, after including death rate (*μ*_*M*_) is:

$$\frac{dM_{2}}{dt}=\underset{\text{M}0\to M1\;\text{by IL}10}{\underset{︸}{\left(f_{2}+\sigma_{M10}\frac{I_{10}}{\zeta_{10}+I_{10}}\right)M_{0}}}+\underset{\text{M}1\to M2\;\text{by TGF-}\beta }{\underset{︸}{\sigma_{M\beta }\frac{I_{\beta }}{\zeta_{\beta }+I_{\beta }}M_{1}}}-\underset{\text{M}2\to M1\;\text{by IFN}\gamma }{\underset{︸}{\sigma_{M\gamma }\frac{I_{\gamma }}{\zeta_{\gamma }+I_{\gamma }}M_{2}}}-\underset{\text{Decay}}{\underset{︸}{\mu_{M}M_{2}}}.$$ (11)

#### Differential equations for T cells

We finally turn to the dynamics of the T cells. The initiation of the inflammatory process involves macrophage activation. Macrophages produce several types of cytokines including IL-12, IFN-γ, IL-4, IL-6, TGF-β, IL-10 and TNF-α, and all these cytokines activate T cells upon binding to their specific receptors. The inflammatory network in IBD involves several auto-activation loops among the four types of T cells:

Th1 cells: As shown in Fig 1(A), the loop of Th1 auto-activation includes IL-12, IFN-γ, and macrophages. IL-12 induces Th1 activation while IFN-γ, produced by Th1 cells, activates macrophages to produce more IL-12 [10, 13].

Th2 cells: The loop of Th2 auto-activation includes IL-4 which leads to Th2 activation while Th2 cells further drive the expression of IL-4 [4, 10, 13]. This positive feedback loop is shown in Fig 1(B).

Th17 cells: Th17 auto-activation involves IL-6, IL-21 and TGF-β. Th17 cells induce production of IL-21 which, together with IL-6 and TGF-β, may further stimulate the activation of Th17 cells [8, 14, 15]. This is shown in Fig 1(C).

Treg cells: The loop of Treg auto-activation includes TGF-β, IL-10 and IL-2. As shown in Fig 1(D), TGF-β and IL-10 trigger Treg activation while Treg cells express TGF-β and IL-10, forming a positive feedback loop in Treg differentiation [4, 10, 17]. We assume that IL-2 is critical for the activation of Treg cells [23].

Aside from activation, the system is regulated by several inhibitory processes (red lines in Fig 1). Treg inhibits the activation of Th1 and Th2 cells [24] while the pairs, Th1-Th2 and Th17-Treg, are mutually antagonistic [9, 25]. IFN-γ produced by Th1 and IL-4 produced by Th2 inhibits Th17 activation [26].

Macrophages activation after encounter of microbial antigen induces Th1 development through direct contact and IL-12 production [10, 13]. Inhibition of IL-12 production by macrophages may explain the ability of IL-10 to suppress Th1 development [27]. Mathematically this is expressed by the following equation:

$$\frac{dT_{1}}{dt}=\sigma_{12}\frac{I_{12}}{\zeta_{12}+I_{12}}\frac{1}{1+I_{10}/\zeta_{10}}M.$$

IL-2 secretion by Th1 cells can further enhance their activation [28]. On the other hand, negative regulatory signals are provided by Th2 and Treg cells [24, 25]. Finally the net rate of Th1 cell production needs to incorporate the activation-induced apoptosis. Hence our original differential equation becomes:

$$\frac{dT_{1}}{dt}=\underset{\text{Cytokine signaling}}{\underset{︸}{\left(\sigma_{12}\frac{I_{12}}{\zeta_{12}+I_{12}}\frac{1}{1+I_{10}/\zeta_{10}}M+\sigma_{2}\frac{I_{2}}{\zeta_{2}+I_{2}}T_{1}\right)}}\underset{\text{Inhibition by Th}2,\;\text{Treg}}{\underset{︸}{\left(\frac{1}{1+T_{2}/\gamma_{2}}\right)\left(\frac{1}{1+T_{r}/\gamma_{r1}}\right)}}-\underset{\text{Decay}}{\underset{︸}{\mu_{1}T_{1}}}.$$ (12)

Similarly, for Th2 we accounted for IL-4 signaling, reciprocal Th2 inhibition by Th1 and Treg programs as well as activation-induced death of Th2 cells [4, 10, 13, 24]:

$$\frac{dT_{2}}{dt}=\underset{\text{Cytokine signaling}}{\underset{︸}{\left(\sigma_{4}\frac{I_{4}}{\zeta_{4}+I_{4}}M\right)}}\underset{\text{Inhibition by Th}1,\;\text{Treg}}{\underset{︸}{\left(\frac{1}{1+T_{1}/\gamma_{1}}\right)\left(\frac{1}{1+T_{r}/\gamma_{r2}}\right)}}-\underset{\text{Decay}}{\underset{︸}{\mu_{2}T_{2}}}.$$ (13)

IL-6 in conjunction with TGF-β activates Th17 cells [8, 14, 15]. IL-21 is also a potent inducer of Th17 differentiation [8, 14, 15]; downregulation of IL-21 expression decreases Th17 cell infiltration in intestinal mucosa of IBD patients. Th17 activation is resisted by both IFN-γ and IL-4 [29]. Since Treg cells are also an inhibitor of Th17 activation [9], the final equation for *T*_*17*_ is:

$$\frac{dT_{17}}{dt}=\underset{\text{Cytokine signaling}}{\underset{︸}{\left[\left(\sigma_{21}\frac{I_{21}}{\zeta_{21}+I_{21}}+\sigma_{6}\frac{I_{6}}{\zeta_{6}+I_{6}}\right)\frac{I_{\beta }}{\zeta_{\beta }+I_{\beta }}\frac{M}{\left(1+I_{\gamma }/\zeta_{\gamma })(1+I_{4}/\zeta_{4}\right)}\right]}}\underset{\text{Inhibition by Treg}}{\underset{︸}{\left(\frac{1}{1+T_{r}/\gamma_{r17}}\right)}}-\underset{\text{Decay}}{\underset{︸}{\mu_{17}T_{17}}}.$$ (14)

Treg cells number is regulated by a dynamic homeostatic process that balances high rates of cell division with apoptosis. These cells have high affinity for IL-2 which is a potent negative regulator of pro-apoptotic signals in Treg cells [23]. Interaction with innate immune cells like macrophages as well as cytokines like TGF-β and IL-10 promotes survival and immunosuppressive activity of this cell population. IBD patients have increased gut production of the proinflammatory cytokine TNF-α which was shown to increase Treg cell apoptosis, and TNF-α blockade can reverse this process, which is also observed in rheumatoid arthritis (RA) patients [30]. Proinflammatory cytokines that drive a Th17 response negatively regulate Treg cell development. Increased Th17 cell density in the gut mucosa of IBD patients corresponds to a reciprocal impairment in the Treg population. Taking in consideration all these positive and negative regulators of Treg cells, we derive the following equation:

$$\frac{dT_{r}}{dt}=\underset{\text{Cytokine signaling}}{\underset{︸}{\left[\left(\sigma_{\beta }\frac{I_{\beta }}{\zeta_{\beta }+I_{\beta }}+\sigma_{10}\frac{I_{10}}{\zeta_{10}+I_{10}}\right)\frac{I_{2}}{\zeta_{2}+I_{2}}\frac{M}{1+I_{\alpha }/\zeta_{\alpha }}\right]}}\underset{\text{Inhibition by Th}17}{\underset{︸}{\left(\frac{1}{1+T_{17}/\gamma_{17}}\right)}}-\underset{\text{Decay}}{\underset{︸}{\mu_{r}T_{r}}}.$$ (15)

### Parameter estimation

#### Macrophages

We assumed that under normal conditions majority of gut macrophages display an M2 like phenotype. The parameters *σ*_*Mγ*_, *σ*_*M10*_, *σ*_*Mβ*_ and *σ*_*Mα*_ represent the macrophage activation related coefficients under the influence of IFN-γ, IL-10, TGF-β and TNF-α cytokines, respectively. Given the assumption that under normal conditions M2 concentration is usually higher than M1, the activation rate of M2 is higher than that of M1; we arbitrarily take *σ*_*M10*_ = 10*σ*_*Mα*_. We also considered that the transition rates between M1 and M2 phenotypes are the same as the activation rate of M2, and take *σ*_*Mβ*_ = *σ*_*M10*_ and *σ*_*Mγ*_ = *σ*_*M10*_.

#### Cytokines

Th1 cells in the inflamed gut likely produce much more IFN-γ than macrophages. Thus we empirically considered the IFN-γ production rate in Th1 cells compared to that of macrophages, to be *ν*_*γ1*_ = 5*ν*_*γM*_. We also assumed a similar production rate of IL-2 (*ν*_*21*_) and IFN-γ (*ν*_*γ1*_) by Th1 cells, so that *ν*_*21*_ = *ν*_*γ1*_.

It was previously shown that Th2 cells at a density of 3 × 10^−2^ g/cm^3^ produce an IL-4 concentration of 15 × 10^−9^ g/cm^3^ [31]. Given *δ*_*4*_ = 349.37 week^−1^ [7, 32] and using the steady-state equation (this is expressed as *ν*_*42*_*T*_*2*_ *− δ*_*4*_ *I*_*4*_ = 0), we get *ν*_*42*_ = 1.75 × 10^−4^ week^−1^. We further assumed that Th2 cells produce more IL-4 than macrophages, and take *ν*_*4M*_ = *ν*_*42*_/3 = 5.83 × 10^−5^ week^−1^.

Mouse models of colitis have indicated that T cell derived IL-10 plays a more important role than the innate immune pool. We assumed that Treg cells produce more IL-10 than macrophages, and we took the IL-10 production rates to be *ν*_*10M*_ = 3.72 × 10^−4^ week^−1^ [29, 33] and *ν*_*10r*_ = 3*ν*_*10M*_.

#### T cells

Maintenance of Th1 cells pool would require the activation and decay coefficients to be of similar magnitude. The coefficient *σ*_*2*_ (representing the IL-2 maximal signal output) was estimated to be 1.23 week^−1^ while *μ*_*1*_ (Th1 degradation rate coefficient) was 1.4 week^-1^.

According to [34], we assume that Th17 activation rates by IL-6 is the same as that by IL-21, so that *σ*_*6*_ *= σ*_*21*_. Similarly, IL-10 and TGF-β contributions to Treg activation/survival rate were taken to be of equal magnitude, so that *σ*_*10*_ *= σ*_*β*_.

#### Other parameters

We take the inhibition of Th2 by Th1 to be *γ*_*1*_ = 1.83 × 10^−1^ g/cm^3^, the inhibition of Treg by Th17 to be *γ*_*17*_ = 3.37 × 10^−1^ g/cm^3^, and assume that the inhibition of Th1 by Th2 is larger, taking *γ*_*2*_ = 5.35 × 10^−2^ g/cm^3^. We also assume that Treg inhibition effect on the other T cells is still somewhat larger, taking *γ*_*r1*_ = *γ*_*r2*_ = *γ*_*r17*_ = 6.06 × 10^−2^ g/cm^3^.

From the data described in the section Data collection and analysis we obtained, in particular, the mRNA concentrations of TNF-α, IL-6 and IL-10 for healthy individuals as well as the master regulators of T cells for healthy individuals. The cytokine concentrations of TNF-α, IL-6 and IL-10 are assumed to be proportional to those of the mRNA concentration, with proportionality parameter λ_c_. In [29], the cytokine concentrations are within 10^−5^–10^-9^g/cm^3^; we take the IL-6 concentration in healthy tissue to be 8.00 × 10^−6^ g/cm^3^. We can then compute λ_c_ and then also the concentrations of TNF-α, IL-10 in healthy tissue. We accordingly get the steady-state concentration of TNF-α to be 9.75 × 10^−6^ g/cm^3^ and of IL-10 to be 1.54 × 10^−6^ g/cm^3^.

The densities of Th1, Th2, Th17 and Treg in healthy tissue are assumed to be proportional to the concentrations of their master regulators with proportionality parameter λ_T_ which needs to be determined; the master regulators are obtained from the mRNA analysis described in the section Data collection and analysis. In addition to λ_T_, there are still six important parameters that need to be determined, namely, *v*_*6M*_, *v*_*α1*_, *σ*_*12*_, *σ*_*4*_, *σ*_*21*_ and *σ*_*β*_ We determine these 7 parameters as follows:

We assume that each half-saturation constant is the same as the steady state and solve the 15 steady-state equations of the system (1)-(15) for the following 15 variables: the 7 parameters defined above, and the 8 steady-state concentrations of IL-2, IL-4, IL-12, IL-21, TGF-β, IFN-γ, M1 macrophages and M2 macrophages.

Solving the steady-state system of 15 algebraic equations, we get the values of the parameters *v*_*6M*_, *v*_*α1*_, *σ*_*12*_, *σ*_*4*_, *σ*_*21*_ and *σ*_*β*_ (as shown in Tables 2 and 3 under “estimated from data”), and the steady-state concentrations of IL-2, IL-4, IL-12, IL-21, TGF-β, IFN-γ, M1 macrophages and M2 macrophages (shown in Table 4). Here we use the fact that the steady-state concentrations of λ_T_Th1, λ_T_Th2, λ_T_Th17 and λ_T_Treg are known from the analysis of the section Data collection and analysis. The half-saturation parameters are listed at the end of Table 3.

**Table 2:** Parameters used in the equations of macrophages and cytokines.

Parameter	Definition	Value	References
**Macrophages**			
*f*_*1*_	Basal activation rate of M1	1 week^-1^	[29] & estimated
*f*_*2*_	Basal activation rate of M2	10 week^-1^	[29] & estimated
*σ*_*Mα*_	Activation rate of macrophages by TNF-α	2.4 week^-1^	[29]
*σ*_*Mγ*_	Transition rate from M2 to M1	24 week^-1^	[29] & estimated
*σ*_*M10*_	Activation rate of macrophages by IL-10	24 week^-1^	[29] & estimated
*σ*_*Mβ*_	Transition rate from M1 to M2	24 week^-1^	[29] & estimated
*M*_*0*_	Density of inactivated macrophages	1.5 x 10^−3^ g/cm^3^	[29, 35]
*μ*_*M*_	Decay rate of macrophages	1 week^-1^	[36]
**Cytokines**			
*ν*_*γM*_	Production rate of IFN-γ by macrophages	8.2 x 10^−6^ week^-1^	[29, 36]
*ν*_*γ1*_	Production rate of IFN-γ by Th1	4.1 x10^-5^ week^-1^	[29, 36] & estimated
*ν*_*4M*_	Production rate of IL-4 by macrophages	5.83 x10^-5^ week^-1^	[31] & estimated
*ν*_*42*_	Production rate of IL-4 by Th2	1.75 x 10^−4^ week^-1^	[31] & estimated
*ν*_*6M*_	Production rate of IL-6 by macrophages	3.63 x 10^−3^ week^-1^	Estimated from data
*ν*_*10M*_	Production rate of IL-10 by macrophages	3.72 x 10^−4^ week^-1^	[29, 33]
*ν*_*10r*_	Production rate of IL-10 by Treg	1.12 x 10^−3^ week^-1^	[33] & estimated
*ν*_*12M*_	Production rate of IL-12 by macrophages	2.65 x 10^−4^ week^-1^	[29, 36]
*ν*_*2117*_	Production rate of IL-21 by Th17	8.05 x 10^−4^ week^-1^	[15]
*ν*_*αM*_	Production rate of TNF-α by macrophages	2.10 x 10^−2^ week^-1^	[29]
*ν*_*α1*_	Production rate of TNF-α by Th1	7.35 x 10^−2^ week^-1^	Estimated from data
*ν*_*βM*_	Production rate of TGF-β by macrophages	5.6 x 10^−9^ week^-1^	[29]
*ν*_*βr*_	Production rate of TGF-β by Treg	3.90 x 10^−8^ week^-1^	[37]
*ν*_*21*_	Production rate of IL-2 by Th1	4.1 x 10^−5^ week^-1^	[29, 36] & estimated
*n*_*2r*_	Production ratio of IL-10 by IL-2	3	Estimated
*δ*_*γ*_	Degradation rate of IFN-γ	29.12 week^-1^	[7, 18]
*δ*_*4*_	Degradation rate of IL-4	349.37 week^-1^	[7, 32]
*δ*_*6*_	Degradation rate of IL-6	29.11 week^-1^	[38]
*δ*_*10*_	Degradation rate of IL-10	116.48 week^-1^	[39]
*δ*_*12*_	Degradation rate of IL-12	8.33 week^-1^	[29]
*δ*_*21*_	Degradation rate of IL-21	63.98 week^-1^	[40]
*δ*_*α*_	Degradation rate of TNF-α	388.15 week^-1^	[41]
*δ*_*β*_	Degradation rate of TGF-β	349.37 week^-1^	[7, 42]
*δ*_*2*_	Degradation rate of IL-2	537.46 week^-1^	[7, 43]

**Table 3:** Parameters used in the equations of T cells and a list of half-saturation constants.

Parameter	Definition	Value	References
**T cells**			
*μ*_*1*_	Decay rate of Th1	1.4 week^-1^	[29, 36]
*μ*_*2*_	Decay rate of Th2	1.4 week^-1^	[29, 36]
*μ*_*17*_	Decay rate of Th17	1.4 week^-1^	[29, 36]
*μ*_*r*_	Decay rate of Treg	1.4 week^-1^	[29, 36]
*σ*_*12*_	Maximum rate of IL-12 signaling	10.93 week^-1^	Estimated from data
*σ*_*2*_	Maximum rate of IL-2 signaling	1.23 week^-1^	[29, 36] & estimated
*σ*_*4*_	Maximum rate of IL-4 signaling	1.94 week^-1^	Estimated from data
*σ*_*21*_	Maximum rate of IL-21 signaling	156.17 week^-1^	[34] & estimated from data
*σ*_*6*_	Maximum rate of IL-6 signaling	156.17 week^-1^	[34] & estimated from data
*σ*_*β*_	Maximum rate of TGF-β signaling	14.02 week^-1^	Estimated from data
*σ*_*10*_	Maximum rate of IL-10 signaling	14.02 week^-1^	Estimated from data
*γ*_*1*_	Constants for Th1 inhibition of Th2	1.83 x 10^−1^ g/cm^3^	Estimated
*γ*_*2*_	Constants for Th2 inhibition of Th1	5.35 x10^-2^ g/cm^3^	Estimated
*γ*_*r1*_, *γ*_*r2*_, *γ*_*r17*_	Constants for Treg inhibition	6.06 x10^-2^ g/cm^3^	Estimated
*γ*_*17*_	Constants for Th17 inhibition	3.37 x10^-1^ g/cm^3^	Estimated
**Half-saturation constants**			
*ζ*_*6*_	Constant for IL-6	8 x 10^−6^ g/cm^3^	The constants are the same as the steady-state concentrations of the cytokines in healthy individuals; in solving for the steady-state equations of (1)-(15)
*ζ*_*α*_	Constant for TNF-α	9.75 x 10^−6^ g/cm^3^	
*ζ*_*10*_	Constant for Il-10	1.54 x 10^−6^ g/cm^3^	
*ζ*_*2*_	Constant for IL-2	6.86 x 10^−8^ g/cm^3^	
*ζ*_*γ*_	Constant for IFN-γ	2.58 x 10^−6^ g/cm^3^	
*ζ*_*12*_	Constant for IL-12	4.90 x 10^−8^ g/cm^3^	
*ζ*_*4*_	Constant for IL-4	9.70 x 10^−9^ g/cm^3^	
*ζ*_*21*_	Constant for IL-21	4.25 x 10^−6^ g/cm^3^	
*ζ*_*β*_	Constant for TGF-β	6.77 x 10^−12^ g/cm^3^	

**Table 4:** Steady-state concentrations of cytokines, macrophages and T cells in a healthy individual.

	Value (g/cm^3^)
M1 macrophage	2.34 x 10^−2^
M2 macrophage	2.50 x 10^−2^
Th1 cell	4.58 x 10^−2^
Th2 cell	1.34 x 10^−2^
Th17 cell	3.37 x 10^−1^
Treg cell	6.06 x 10^−2^
IFN-γ	2.58 x 10^−6^
IL-2	6.86 x 10^−8^
IL-4	9.70 x 10^−9^
IL-21	4.25 x 10^−6^
IL-6	8.00 x 10^−6^
TNF-α	9.75 x 10^−6^
IL-10	1.54 x 10^−6^
TGF-β	6.77x 10^−12^
IL-12	4.90 x 10^−8^

For completeness we listed in Table 4 all the steady-state concentrations of cytokines, macrophages and T cells; this list partially overlaps with Table 3.

### Parameter sensitivity analysis

Since the model is highly complicated with a lot of parameters and some parameters are estimated roughly from data, we performed sensitivity analysis to determine the robustness of the simulation results and effect of the parameters on the concentrations of Th1 and Th2 cells which are used to determine the types of IBD patients. In our parameter analysis, we focused on nine parameters, *σ*_*Mα*_, *σ*_*M10*_, *σ*_*12*_, *σ*_*2*_, *σ*_*4*_ *σ*_*21*_, *σ*_*6*_, *σ*_*β*_ and *σ*_*10*_, which are the activation rates of macrophages and T cells by different types of cytokines and are the most significant in the disease dynamics.

**Fig 2:**
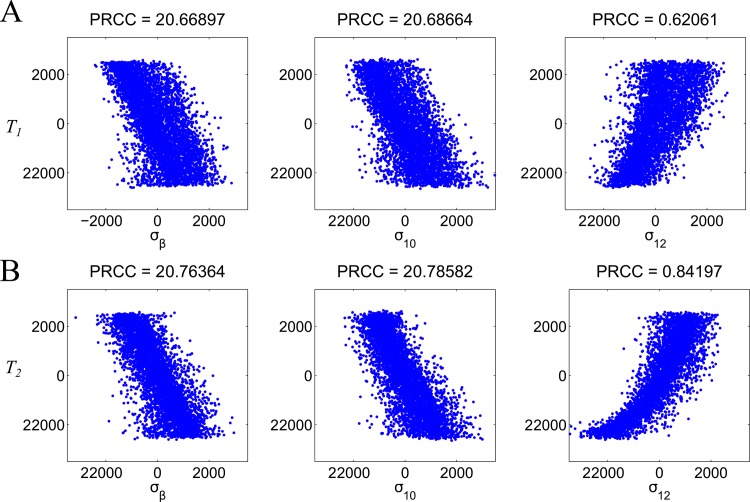
**Scatter plots of rank transformed *T***_**1**_ **(A) and *T***_**2**_ **(B) after 100 weeks versus some rank transformed parameters with statistically significant correlation (|PRCC|>0.5 and p-value <0.01).** The title of each subplot shows its PRCC value.

We applied the method of Partial Rank Correlation Coefficient (PRCC) [44] for our sensitivity analysis. We ran 5000 simulations in which all the nine parameters are varied according to Latin hypercube sampling with the range of ±20% perturbation around the parameter values obtained for healthy individuals. The results of the sensitivity analysis are summarized in Table 5. Fig 2 shows the scatter plots of rank transformed *T*_1_ (Fig 2A) and *T*_2_ (Fig 2B) after 100 weeks (solution close to steady state) versus the rank transformed parameters with significant correlation (|PRCC|>0.5) and p-value (p<0.01); the title of each subplot shows its PRCC value.

**Table 5:** PRCC values of some parameters for the outputs *T*_1_ and *T*_2_.

	PRCC for *T*_1_	PRCC for *T*_2_
*σ*_*Mα*_	0.3298*	-0.0190
*σ*_*M10*_	0.6264*	-0.1448*
*σ*_*12*_	0.6206*	0.8420*
*σ*_*2*_	0.2257*	0.4404*
*σ*_*4*_	-0.3123*	-0.4336*
*σ*_*21*_	0.2756*	0.3192*
*σ*_*6*_	0.4181*	0.4954*
*σ*_*β*_	-0.6690*	-0.7636*
*σ*_*10*_	-0.6866*	-0.7858*

* Denotes significant PRCC values (p-value <0.01).

All the nine parameters have significant PRCC values either with *T*_1_ or with *T*_2_. Among the nine parameters, we find that the statistically significant PRCC values with both *T*_1_ and *T*_2_ are *σ*_*12*_, *σ*_*β*_ and *σ*_*10*_. The parameters *σ*_*β*_ and *σ*_*10*_ are negatively correlated to *T*_1_ and *T*_2_: the two parameters are the activation rates of Treg cells which inhibit Th1 and Th2 activations. This result is consistant with the observation that the abnormal levels of T cells usually arise from abnormal regulation of Th1 and Th2 cells by Treg cells [7]. On the other hand, *σ*_*12*_ is positively correlated to *T*_1_ and *T*_2_: as the parameter increases, the amount of Th1 cells increases and thus Treg cell regulation on Th1 and Th2 cells decreases through increased TNF-α inhibtion produced by Th1 cells. Note that although Th1 and Th2 cells inhibit each other, reduced Treg inhibtion has a larger effect than the mutual inihbition between Th1 and Th2 cells. The roles of these three parameters (*σ*_*12*_, *σ*_*β*_ and *σ*_*10*_) on different types of IBD will be discussed in the Result section later.

### Data collection and analysis

#### Human subjects and tissue biopsy collection

Colon biopsies from healthy controls and patients with Inflammatory Bowel Diseases were obtained from a de-identified tissue bank. The tissue bank is Host Modifiers of Inflammatory Bowel Diseases Phenotypes and IRB protocol number is 06-0270-F1V. The Institutional Review Board at the University of Kentucky approved the tissue bank protocol. Banked, normal biopsies were obtained from healthy controls that underwent screening colonoscopy. IBD patients had an established diagnosis of Crohn’s disease involving the colon and were off immunosuppressant treatment at the time of the endoscopic procedure. Biopsies were obtained inflamed but not ulcerated colonic mucosa. All measures were derived from mucosal biopsies. No blood samples were utilized in our experiment. Patient characteristics are shown in Table 6.

**Table 6:** Baseline characteristics for patients with IBD-Crohn’s Disease.

Total number	N = 58
Gender	
Female	27(46.55%)
Male	31(53.45%)
Location	
Upper GI	0(0%)
Ileal	0(0%)
Ileo-colonic	47(81.03%)
Colonic	11(18.97%)
Perianal	6(10.34%)
Behavior	
Inflammatory	41(70.68%)
Stricturing	14(24.13%)
Fistulizing	3(5.17%)
Current immunosuppressant treatment	0(0%)
Former use of immunomodulator	27(46.55%)
Former use of biologic therapy	19(32.75%)

Cytokine profile data tends to be affected by the contamination of epithelial cells that cannot produce typical Th1/Th2 cytokines. Nevertheless all biopsies are expected to have a similar content of epithelial cells: we used the same standard biopsies forceps, applied en-face orientation, and for all IBD patients the biopsy was performed at the area abutting ulceration. If anything, biopsies from IBD patients would have been expected to contain less epithelial cells, due to surface erosion. Since control and IBD biopsies contained epithelial cells the differences in immune markers are expected to be representative of the inflammatory process.

#### mRNA analysis

Frozen biopsies were placed in 600μl lysis buffer (MagNA Pure Compact RNA Isolation Kit) at room temperature for 10 min. Biopsies were disrupted using a MagNA Lyser instrument (Roche, Basel Switzerland) and beads: 1^st^ spin @ 7000x for 40 sec and 2^nd^ spin @6000x for 30 sec. Tubes were placed in the instrument cooling block for 15 min and then centrifuged briefly to pellet the debris. The supernatant containing total RNA was then purified by MagNA Pure Compact RNA Isolation Kit (Roche) protocol with elution volume of 50ul. For control RNA quality and quantity were determined with a spectrophotometer (NanoDrop Technologices, Wilmington, DE).

#### Gene expression analysis

Total mRNA (15μl) were analyzed using the nCounter Master Kit from NanoString Technologies (NanoString Technologies, Inc Seattle, WA). Gene Expression CodeSets of our interest for the nCounter Analysis System were preordered from NanoString Technologies, Inc (www.nanostring.com). The nCounter Analysis System is an integrated system comprised of a fully-automated Prep Station, a Digital Analyzer, and the CodeSet (molecular barcodes) reader.

## Results

In the sequel we shall use the following abbreviations:

*T*_*1ssh*_: the mean value of the Th1 master regulator T-bet of healthy individuals
*T*_*1ss*_: the value of the Th1 master regulator T-bet of IBD patient
*T*_*2ssh*_: the mean value of the Th2 master regulator GATA-3 of healthy individuals
*T*_*2ss*_: the value of the Th2 master regulator GATA-3 of IBD patient

We obtained gut mucosal samples from healthy individuals and IBD-Crohn’s patients and measured the mRNA expression for cytokines IL-6, IL-10, TNF-α and transcription factors T-bet, GATA3, RORγt and Foxp3. Based on the observed values we were able to divide the patients into four categories:

***Type 1*.** *T*_*1ss*_ > *T*_*1ssh*_, *T*_*2ss*_ < *T*_*2ssh*_
***Type 2*.** *T*_*1ss*_ < *T*_*1ssh*_, *T*_*2ss*_ > *T*_*2ssh*_
***Type 3*.** *T*_*1ss*_ > *T*_*1ssh*_, *T*_*2ss*_ > *T*_*2ssh*_
***Type 4*.** *T*_*1ss*_ < *T*_*1ssh*_, *T*_*2ss*_ < *T*_*2ssh*_

For example, if an IBD patient had a T-bet level higher than *T*_*1ssh*_ and a GATA-3 level lower than *T*_*2ssh*_, this patient is classified to Type 1 group. There are totally 58 patients: 7 in Type 1, 18 in Type 2, 17 in Type 3 and 16 in Type 4. The deviations of mRNA fold expression of IBD patients from healthy individuals are shown in Table 7.

**Table 7:** Fold changes of the cytokine and T cell concentrations obtained from the clinical data and the simulations in different types of diseases.

Type of diseases	# of cases	IL-6	IL-10	TNF-α	T-Bet	Gata3	RORγt	Foxp3	TGF-β*	IFN-γ*
Th1↑ Th2↓	7	↓-93%	↓-51%	↓-31%	↑+50%	↓-22%	↑+50%	↓-71%	↓-56%	↓-99%
Th1↓Th2↑	18	↓-74%	↓-15%	↓-31%	↓-29%	↑+50%	↓-27%	↑+1%	↓-6%	↓-92%
Th1↓Th2↑	18	↓-74%	↓-15%	↓-31%	↓-29%	↑+50%	↓-27%	↑+1%	↓-6%	↓-92%
Th1↑Th2↑	17	↑+35%	↑+44%	↑+47%	↑+110%	↑+75%	↓-14%	↑+70%	↑+19%	↑+481%
Th1↓Th2↓	16	↓-83%	↓-34%	↓-45%	↓-46%	↓-40%	↓-11%	↓-42%	↓-20%	↓-95%

* The values of TGF-β and IFN-γ are predicted by the model.

Our next step was to determine the values of coefficients that define the production rate of individual cytokines and activation of master regulators. These theoretical parameter variations (cytokine production, T cell activation), in each of the four types, were determined using the steady-state solution of our mathematical model and are shown in Table 7. We assumed that the blood sample data reflect the tissue data (by the same proportionality parameter), and that the disease state occurred at the steady state when some of the production/activation rates related to T cells were deregulated, either increased or decreased. Here we consider the variations of the following parameters, *ν*_*g1*_, *ν*_*α1*_, *ν*_*βr*_, *ν*_*10r*_, *σ*_*12*_, *σ*_*4*_, *σ*_*21*_, *σ*_*6*_, *σ*_*β*_ and *σ*_*10*_ (Table 8).

**Table 8:** Simulation results: Parameter variations in different types of diseases.

Type of diseases	*ν*_*γ1*_	*ν*_*α1*_	*ν*_*βρ*_	*ν*_*10ρ*_	*σ*_*12*_	*σ*_*4*_	*σ*_*21*_	*σ*_*6*_	*σ*_*β*_	*σ*_*10*_
	coefficient of IFN-γ by Th1	coefficient of TNF-α by Th1	coefficient of TGF-β by Treg	coefficient of IL-10 by Treg	activation of Th1 cells	activation of Th2 cells	activation of Th17 cells	activation of Th17 cells	activation of Treg cells	activation of Treg cells
Th1↑Th2↓	↓-99.6%	↓-50%	↑+35%	↑+35%	↑+298%	↓-32%	↑+49%	↑+49%	↓-50%	↓-50%
Th1↓Th2↑	↓-90%	↑+4%	↓-10%	↓-10%	↑+81%	↑+25%	↓-13%	↓-13%	-0%	-0%
Th1↑Th2↑	↑+194%	↓-29%	↓-30%	↓-30%	↑+255%	↑+122%	↑+298%	↑+298%	↑+17%	↑+17%
Th1↓Th2↓	↓-92%	↑+11%	↑+32%	↑+32%	↑+5%	↓-48%	↓-28%	↓-28%	↓-18%	↓-18%

In type 1 group the production rate of Th1 cytokines, IFN-γ and TNF-α_,_ was decreased while the anti-inflammatory IL-10 was increased. Nevertheless the signal strength of IL-12, IL-21 and IL-6 favored activation of Th1 and Th17 pathways. On the other hand, decreased activation of Treg by TGF-β and IL-10 was able to offset their increased production rate.

In type 2 group, despite an increase in TNF-α production rate and IL-12 signal strength, the lack of IL-21 and IL-6 signal strength coupled with increased IL-4 signal strength tipped the balance in favor of Th2 response.

Type 3 group was the only one with a robust IFN-γ production rate. Interestingly we no longer observed the reciprocity between the Th1, Th2, Th17 and Treg activation rates, as the signal strength for IL-12, IL-4, IL-21, IL-6, TGF-β and IL-10 were increased across the board.

Type 4 group was characterized by a global decrease in signal strength and thus lack of T cell activation even though this group had the highest production rate of TNF-α

**Fig 3:**
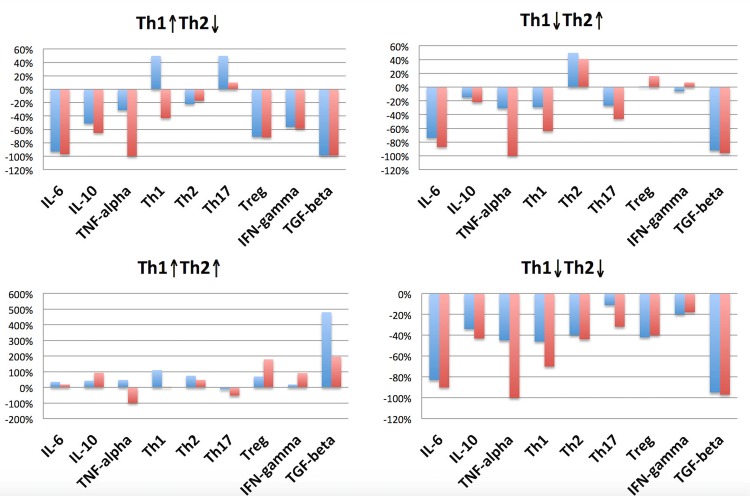
Simulation results: fold changes of the cytokine and T cell concentrations when TNF-α is completely blocked in different types of diseases. Blue bars represent the results of pre-treatment; red bars represent the results of post-treatment with TNF-α blockage.

Currently, anti-TNF antibodies represent the main group of biologic agents for the treatment of IBD. Therefore we simulated the TNF-α blockade in our mathematical model. For modeling the TNF-α blockade, we fix the concentration of TNF-α (*I*_α_) to be zero and calculate the change of other variables at the steady-state concentrations. Fig 3 shows the predicted mRNA fold change in cytokines and T cell master regulators. In type 1 group, TNF-α blockade had the most profound effect on Th1 (93%) and Th17 (40%) master regulators. In the third group our model predicted a significant decrease in Th1 (105%) and increase in Treg (108%). In the second and fourth group the decrease in both Th1 (35%, 34%) and Th17 (19%, 21%) master regulators was modest whereas Treg was virtually unaffected (1%, 2%).

## Conclusions and Discussion

Inflammatory Bowel Diseases are characterized by a deregulated immune response toward gut microbiota. A simplistic characterization of disease phenotype divides patients into either Crohn’s Disease or Ulcerative Colitis. Nevertheless, if we take into account the individual variations in genetic background, microbiome, environmental exposure, nutrition, and life-style, we can foresee multiple immune phenotypes. The development of current biological therapies in CD mirrors our understanding of immune regulatory pathways. Current Montreal classification distinguishes phenotypes based on anatomical and clinical criteria. Unfortunately this will not help identify individual immune phenotype relevant to a particular biologic treatment. Pretreatment knowledge of the relevant gut mucosal immune dysfunction in a given patient would significantly improve the risk/benefit balance and open the way toward personalized medical care.

Patients with inflammatory bowel diseases have elevated levels of circulating and gut mucosal cytokines [1]. Downstream signaling from these inflammatory mediators, activate transcription factors T-bet, GATA3, Foxp3 and RORγt [3]. A complex network of regulatory feedback loops involving these cytokines and their targets, are responsible for the polarization of naïve T cells into specific T helper cells: Th1, Th2, Th17 and Treg [3, 4].

In this study we developed a mathematical model of differential equations to describe immune regulatory pathways in patients with CD. The output of these differential equations was dependent on the relative mRNA expression of specific immune system targets.

Patient stratification based on calculated Th1 and Th2 immune cell activation, relative to healthy controls, identified 4 distinct immune phenotypes. These were further characterized based on cytokine production as well as the mRNA expression of the Th1/Th2/Th17 and Treg master regulators.

While it is tempting to analyze individual cytokines when predicting response to a particular biological treatment, the overall immune response is a resultant of opposing factors. The relative magnitude of the end state compared to healthy controls or pre/post treatment status may offer a way to predict response to a particular biologic treatment. We performed an *in-silico* simulation of TNF-α blockade for each of the 4 immune phenotypes identified by our mathematical algorithm. Globally the largest impact was seen in type 3 group (Th1^high^/Th2^high^) where a diminished Th1 and Th17 activation was predicted along with a reciprocal increase in Treg and TGF-β. Therefore this group appeared to have the best immunologic outcome by blocking the TNF-α. Our findings through mathematical modeling mirror the observations in patients with IBD, where Infliximab treatment down-regulates IL-17 expression in the gut mucosa and promotes healing [45]. On the other hand, type 4 was more consistent with a global immunosuppressed status, and anti-TNF blockade further magnified this changes relative to healthy controls. Our study included samples from symptomatic and/or with evidence of disease activity (abnormal CRP, Calprotectin, anemia) CD patients. All patients were off immunosuppressive therapy at the time of endoscopy for at least 3 month. 8 (50%) of type 4 group patients had previously experienced a failure to at least one biologic treatment. Thus type 4 group (Th1^Low^/Th2^Low^) should be a candidate for immune stimulatory therapy rather than further immunosuppression. Also, importantly, the paradoxical presence of clinical and endoscopic activity in this group underscores the importance of global analysis rather than individual immune mediator analysis. In conclusion we propose that mathematical modeling of immune regulatory networks in patients with IBD may allow identifying distinct groups with high relevance to biological therapies. Based on the results of the in-silico simulation of TNF blockade, a multi-pronged approach aimed at several distinct pathways might be superior to biologic monotherapy. In certain cases (type 4) immune activation rather than suppression might be recommended.

Although parameters (coefficients for differential equations) were obtained from other models, they are representative of a chronic inflammatory process and thus relevant to IBD. Sarcoidosis and Lupus (models referenced in our study) also share genetic susceptibility loci with Crohn's disease. Ultimately, validation and refinement of the mathematical parameters will require a prospective approach in IBD patients. Our current model is supposed to provide a basis for such future studies.

We acknowledge that more cytokines could have been included in the present study. Coefficients utilized in the present mathematical model were also derived from prior models of inflammation, which did not include IL-5, IL-13, IL-17 and IL-22. Naïve T helper cells can be induced to differentiate towards T helper 1 (Th1), Th2, Th17 and regulatory (Treg) phenotypes according to the local cytokine milieu. Stable expression of specific transcription factors, T-bet (Th1), GATA-3 (Th2), Foxp3 (Tregs) and RORγt (Th17) will ultimately represent the net effect of the combine cytokine production. Thus the inclusion of all the master regulators relevant to the T cell polarization is expected to reflect the tissue status of these pathways. This potential extension of the modeling with inclusion of the extra cytokines should be considered in future work when more patients data become available.

Our present study represents a snapshot of CD patients evaluated for assessment of disease activity. We intentionally excluded patients with recent exposure to immunosuppressant therapy to avoid a direct influence of the chosen markers. Nevertheless the purpose of the current study was to establish a mathematical model based on IBD patient samples that will serve as a basis for prospective studies.
