# Correction: Inflammatory Bowel Disease: How Effective Is TNF-α Suppression?

**DOI:** 10.1371/journal.pone.0170865

**Published:** 2017-02-02

**Authors:** The PLOS ONE Staff

There is an error in the image for Fig 1. Please see the correct Fig 1 here. The publisher apologizes for the error.

**Fig 1:**
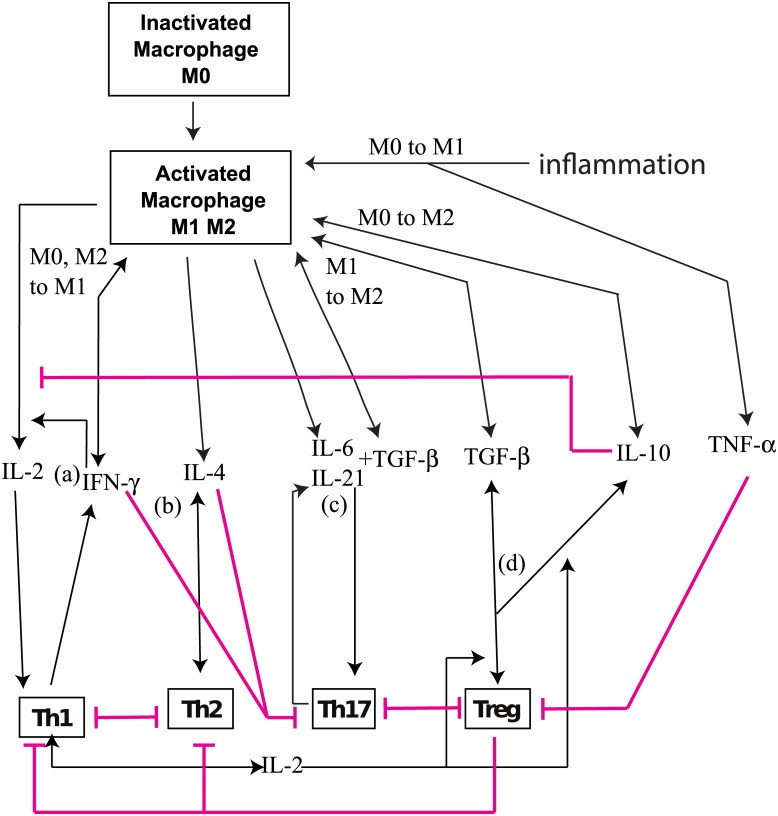
Schematic diagram of immune system with application to inflammatory bowel disease (IBD).

## References

[1] Lo W-C, Arsenescu V, Arsenescu RI, Friedman A (2016) Inflammatory Bowel Disease: How Effective Is TNF-α Suppression? PLoS ONE 11(11): e0165782 doi: 10.1371/journal.pone.0165782 27824890 10.1371/journal.pone.0165782 PMC5100971

